# Circulating microRNAs as Biomarkers of Brain Metastases in Lung Cancer: A Pilot Study

**DOI:** 10.3390/jcm15031083

**Published:** 2026-01-29

**Authors:** Karol Marschollek, Maciej Powierża, Dorota Kujawa, Monika Kosacka, Maciej Majchrzak, Anna Brzecka-Bonnaud, Aneta Kowal, Sławomir Budrewicz, Łukasz Łaczmański, Anna Pokryszko-Dragan

**Affiliations:** 1 Clinical Department of Neurology, University Centre of Neurology and Neurosurgery, Faculty of Medicine, Wroclaw Medical University, Borowska 213, 50-556 Wrocław, Poland; slawomir.budrewicz@umw.edu.pl (S.B.); anna.pokryszko-dragan@umw.edu.pl (A.P.-D.); 2 Laboratory of Genomics and Bioinformatics, Hirszfeld Institute of Immunology and Experimental Therapy, Polish Academy of Sciences, Weigla 12, 53-114 Wrocław, Poland; maciej.powierza@hirszfeld.pl (M.P.); dorota.kujawa@hirszfeld.pl (D.K.); lukasz.laczmanski@hirszfeld.pl (Ł.Ł.); 3 Department of Pulmonology and Lung Cancer, Faculty of Medicine, Wroclaw Medical University, Grabiszyńska 105, 53-439 Wrocław, Poland; monika.kosacka@umw.edu.pl (M.K.); anna.brzecka@umw.edu.pl (A.B.-B.); 4 Thoracic Surgery Clinic, University Center for General and Oncological Surgery, Wroclaw Medical University, Grabiszyńska 105, 53-439 Wrocław, Poland; maciej.majchrzak@umw.edu.pl; 5 Lower Silesian Center of Oncology, Pulmonology and Hematology, Hirszfelda 12, 53-413 Wrocław, Poland; aneta.kowal@dcopih.pl

**Keywords:** lung cancer, NSCLC, miRNA, brain metastases

## Abstract

**Background/Objectives**: There is an ongoing search for reliable biomarkers of lung cancer (LC) and its progression, including nervous system involvement. MicroRNAs (miRNAs) play a crucial role in the regulation of gene expression and represent a promising focus of investigation in this field. The aim of this study was to assess the profile of miRNA expression in patients diagnosed with lung cancer, with or without brain metastases. **Methods**: This study comprised 13 patients diagnosed with non-small cell lung cancer (mean age 64.8 years, 61.5% females): 6 with brain metastases (LC + BM) and 7 without them (LC), and a control group of 6 healthy volunteers (HC). The expression levels of 179 miRNAs were assessed and compared between the study groups using quantitative reverse-transcription PCR (qRT-PCR). **Results**: In LC + BM subgroup, two miRNAs were found to be downregulated in comparison with HC: miR-409-3p (logFC = −17.42, *p* = 0.029) and miR-485-3p (logFC = −17.30, *p* = 0.026). An exploratory, probe-based feature-ranking analysis identified eleven miRNAs that were repeatedly selected across the resampling runs: miR-363-3p, miR-210-3p, miR-194-5p, miR-409-3p, miR-22-3p, miR-2110, miR-326, miR-485-3p, miR-223-5p, miR-16-2-3p, and miR-139-5p. Among these, miR-363-3p, miR-210-3p, and miR-194-5p exhibited the highest empirical stability. Predictive modeling was subsequently evaluated using a fully nested cross-validation framework in which feature selection and model training were repeated within each training fold. Under this stringent evaluation, the classification performance was close to chance across all the evaluated algorithms, indicating a limited predictive utility of the identified miRNAs for distinguishing patients with and without brain metastases in the present dataset. **Conclusions**: Notable differences in miRNA expression profiles were revealed for the patients with brain metastases from lung cancer, suggesting the role of the selected miRNAs in cancer metastasis to the CNS. However, while our analysis provides exploratory insights, the findings should be interpreted with caution and require validation in larger, independent cohorts before any clinical or translational implications can be established.

## 1. Introduction

Lung cancer is the most common malignancy and the leading cause of cancer-related deaths in the general population. According to epidemiological data, in 2022, almost 2.5 million new cases of lung cancer were diagnosed worldwide, and 1.8 million deaths due to it were recorded, which constituted almost 1/5 of overall mortality [1]. Lung cancer is characterized by a high propensity for metastatic spread, which contributes significantly to its poor prognosis and remains a major clinical challenge in the management of the affected patients. In one large, population-based study, as many as 56% of the patients with lung cancer had metastases diagnosed between diagnosis and death, the most common location of which were metastases to the central nervous system (CNS), detected in 39% of patients [2]. Apart from the adverse effects on the prognosis, these CNS metastases have a profound and devastating impact on the patients’ condition due to symptoms of neurological deficit and raised intracranial pressure. Moreover, in a large percentage of cases, the presence of distant metastases is already observed at the initial diagnosis of lung cancer. In both non-small cell lung cancer (NSCLC) and small cell lung cancer (SCLC), approximately 10% of patients are diagnosed with CNS metastases at the first clinical presentation of a neoplasm [3,4].

From a clinical point of view, the early detection of neoplastic metastasis to the CNS is extremely important. He et al. [5] reported a significantly longer overall survival (OS) in patients who received brain surgery and/or radiotherapy before surgical removal of the primary lung tumor. It is also known that carriers of oncogenic driver mutations, primarily mutations in the Epidermal Growth Factor Receptor (EGFR) and Anaplastic Lymphoma Kinase (ALK) rearrangement genes, are more susceptible to the occurrence of brain metastases [6,7], and the implementation of targeted treatment significantly improves the prognosis and the intracranial progression-free survival [8,9].

The identification of patients with lung cancer who are at higher risk for CNS metastases may therefore influence therapeutic decisions. Because of that, simple and easily accessible biomarkers are constantly sought, which would allow for the individualization of patient screening in this direction. Thanks to the development of advanced molecular techniques, particular attention is directed toward the use of “liquid biopsy”, enabling the evaluation of molecules circulating in the peripheral blood, such as circulating tumor DNA (ctDNA) or miRNAs [10].

MicroRNAs (miRNAs) are single-stranded, short, noncoding RNAs, typically around 21 nucleotides in length. As a part of miRNA-induced silencing complex (miRISC) they bind to the 3′ untranslated region (3′-UTR) of target messenger RNAs, acting as post-transcriptional regulators of gene expression [11]. miRNAs are believed to play a significant role in the development and progression of cancer, as they can modulate the expression of both oncogenes and tumor suppressor genes [12].

An assessment of the expression of specific miRNAs has already been applied in lung cancer. It has been shown that specific panels of miRNAs can serve as diagnostic biomarkers, enabling the distinction of malignant tumors from benign ones [13,14] or differentiating histopathological types of lung cancer [15]. In addition, miRNAs have been assessed as potential markers of disease progression [16,17] and of treatment response [18]. Despite the progress in understanding of the role of miRNAs in the development of lung cancer, there is still a lack of consistent data and firm validation of these biomarkers in large cohorts of patients, which limits the possibility of their routine use in clinical practice.

In particular, little is known about the role of an miRNA expression profile as a potential marker of CNS involvement, which seems a significant research gap in the context of lung cancer metastasis. Therefore, we made an attempt to address this topic in our investigation.

The aim of this study was to assess the profile of miRNA expression in patients diagnosed with lung cancer, with or without CNS metastases.

## 2. Materials and Methods

### 2.1. Study Groups

The subjects were recruited from patients hospitalized in the Departments of Pulmonology and Lung Cancer, Thoracic Surgery and Neurology at Wroclaw Medical University and in the Lower Silesian Centre of Oncology, Pulmonology and Hematology, between June 2022 and December 2023.

The LC group comprised the patients with histologically confirmed non-small cell lung cancer (NSCLC) without radiologically detectable brain metastases at the assessment. The second group (LC + BM) consisted of histologically confirmed NSCLC patients with concomitant brain metastases. All brain metastases were classified as synchronous, defined as detected within 3 months of the initial diagnosis. The presence of brain metastases was assessed by magnetic resonance imaging (MRI) or contrast-enhanced computed tomography (CT) of the head. The following exclusion criteria were applied: active infection at the time of admission, any concomitant chronic neurological or inflammatory/autoimmune disorders, history of any malignancies in the past 10 years, and lack of recent (since the diagnosis of lung cancer) brain imaging results (CT or MRI).

Finally, 7 patients were qualified for the LC group and 6 for LC + BM group. All these patients were diagnosed with NSCLC. Then, 7 healthy volunteers were recruited for the control group (HC), selected to match the patient groups in terms of age and sex distribution; 1 was excluded during quality control, resulting in 6 controls included in the final analysis.

### 2.2. Data Collection and Analysis

Demographic and clinical data were extracted from medical charts of each patient.

Samples of venous blood were collected from all participants at admission, centrifuged and stored at −20 °C until use. All samples were analyzed under the same laboratory conditions at the same time.

For molecular analysis, serum samples from study groups were processed for miRNA isolation using the miRCURY RNA Isolation Kit for Biofluids (Exiqon, Vedbæk, Denmark), following the manufacturer’s protocol. Subsequently, a complementary DNA (cDNA) library was acquired.

In order to assess the efficacy of RNA extraction, the internal spike-in controls UniSp2 and UniSp4 were used. The efficiency of cDNA synthesis was evaluated using UniSp6. A quantification cycle (Cp) value exceeding 37 for any of these controls was considered indicative of poor sample quality or low yield. Hemolysis and the presence of PCR inhibitors in serum samples were evaluated by measuring the expression levels of miR-451 and miR-23a. Samples with ΔCp(23a–451) greater than 7 were considered unreliable due to significant hemolysis.

The expression of 179 serum miRNAs was analyzed using the miRCURY LNA™ Serum/Plasma Focus PCR Panel (Exiqon), which utilizes Locked Nucleic Acid (LNA™) technology. Quantitative real-time PCR (qPCR) was performed using a CFX95 Real-Time PCR System (Bio-Rad, Hercules, CA, USA) on 96-well plates. The reaction conditions specified by the manufacturer were used.

Relative expression levels of miRNAs in cancer patient samples were calculated using the comparative Cp method:

ΔCp = Cp_sample_ − Cp_control_

To quantify the difference in expression, the Log Ratio (LR) was calculated as the base 2 logarithm of the fold change. An LR value greater than 1 was interpreted as upregulation, and an LR less than −1 as downregulation. Cp_control_ represents the mean Cp across the control group.

The miRNA analysis was conducted according to the validated and previously published method [19].

All the procedures were performed in accordance with the Declaration of Helsinki and its further amendments. All participants provided written informed consent prior to inclusion in this study. The project of this study was approved by the Wroclaw Medical University Bioethics Committee (approval no. KB-918/2021).

### 2.3. Statistical Analysis

All analyses were performed using R statistical software (Version 4.3.2). In addition to base R functions, the caret package was employed for data preprocessing, model training, and construction of confusion matrices. The pROC, ggplot2, and dplyr packages were used for result visualization and data handling.

First, observations from the HC and LC groups were aggregated into a single category to construct a contrast aimed at identifying miRNAs potentially associated with metastatic progression. Variables with near-zero variance were removed as an unsupervised preprocessing step.

Given the high-dimensional structure of the data, an exploratory, filter-based feature selection procedure was applied within a nested cross-validation framework. For each feature, a univariate Wilcoxon–Mann–Whitney test was computed and used solely as a ranking score, not for confirmatory hypothesis testing; therefore, the resulting *p*-values were not interpreted as measures of statistical significance.

Feature selection was performed using a probe-based procedure [20]. In each iteration, 50 probe variables were generated by randomly selecting an original feature and permuting its sample values to destroy any association with the outcome while preserving its marginal distribution. Original and probe variables were jointly ranked by their Wilcoxon scores, and the largest top-ranked set for which the running ratio of probes to original variables did not exceed 0.1 was retained.

To assess selection stability, the entire probe-based selection procedure was repeated across 100 random seeds within each training fold. Selection frequencies were computed for each original feature across repetitions, and features with a stability of at least 0.45 were retained. Importantly, this entire feature-selection workflow (including probe generation, ranking, stability assessment, and thresholding) was executed independently within each leave-one-out cross-validation (LOOCV) training fold, using only the training data for that fold. The held-out sample was not used at any stage of feature selection.

After feature selection, predictor variables were centered and scaled using statistics computed from the training fold only, and the same transformation was applied to the corresponding held-out sample.

Three classification algorithms, logistic regression with elastic net regularization (GLMNET), linear support vector machine (SVM), and random forest (RF), were evaluated. For each algorithm, hyperparameters were tuned within the training fold using an inner LOOCV procedure. Model performance was assessed using an outer LOOCV loop, where predictions were generated for each held-out sample from models trained without access to that sample.

Predictive performance was summarized using the area under the receiver operating characteristic curve (AUC), computed from the outer-fold predictions. Uncertainty in AUC estimates was quantified using bootstrap confidence intervals based on resampling of subjects.

Significance level was established as α  =  0.05.

## 3. Results

### 3.1. Study Population

One of the samples in the control group was excluded due to the insufficient quality of the microarray results; therefore, the final analysis was conducted on 19 patient samples. Serum samples from 19 subjects were included in the final analysis, of which 12 were women (63.2%). The mean age of the patients with LC was 64.8 years.

There were no significant differences in age or gender distribution between the analyzed groups. The basic demographic and clinical data of the participants are presented in Table 1.

### 3.2. miRNA Expression Analysis

**Figure 1:**
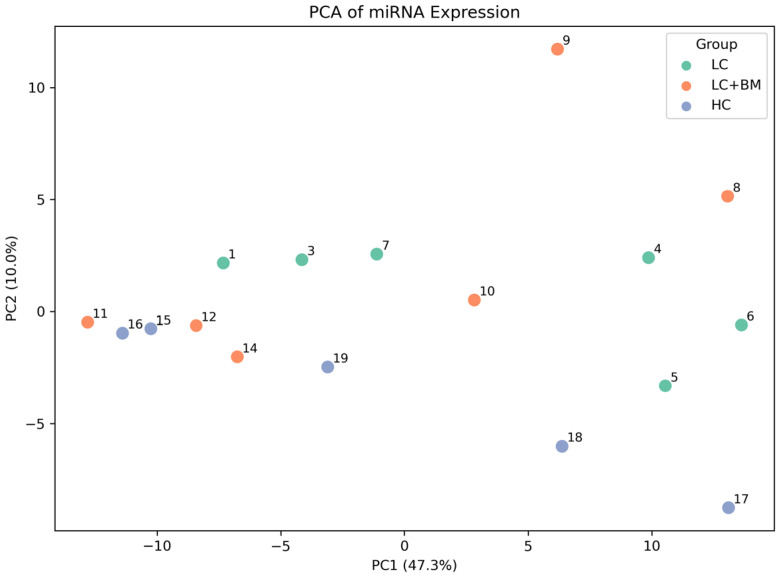
A principal component analysis map for the analyzed groups: lung cancer patients without brain metastases (green), lung cancer patients with brain metastases (orange), healthy controls (blue). LC—lung cancer; BM—brain metastasis; HC—healthy control.

**Figure 2:**
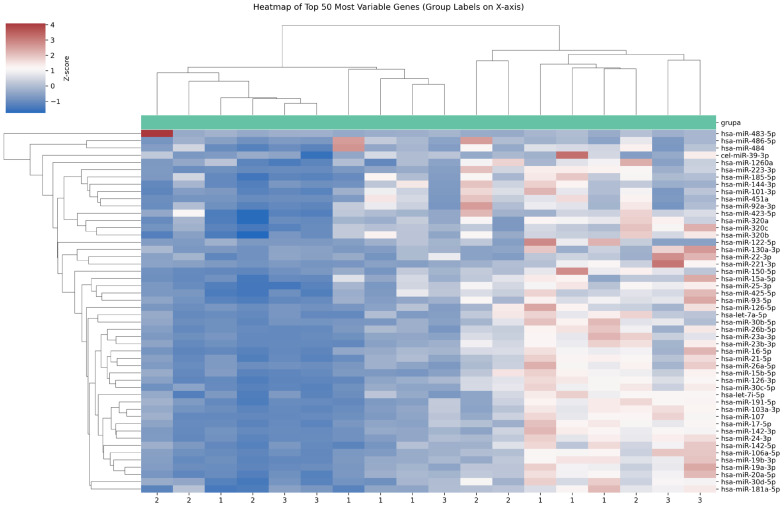
Heatmap of miRNA expression in the analyzed groups. (1) lung cancer patients without brain metastases, (2) lung cancer patients with brain metastases, (3) healthy controls.

The expression levels of the 179 miRNAs were measured in the study samples. Principal component analysis mapping and hierarchical unsupervised clustering analysis were performed to visualize the expression patterns of the miRNAs in the patients and are displayed in Figure 1 and Figure 2.

The miRNA expression profiles were compared between the groups. The exploratory analysis showed a significant downregulation of nine miRNAs in the patients in the LC group compared to the HC group: miR-328-3p (logFC = −16.51, *p* = 0.005), miR-92a-3p (logFC = −9.46, *p* = 0.008), miR-101-3p (logFC = −14.57, *p* = 0.021), miR-451a (logFC = −8.18, *p* = 0.027), miR-374a-5p (logFC = −15.74, *p* = 0.028), miR-100-5p (logFC = −17.61, *p* = 0.036), miR-197-3p (log FC = −15.71, *p* = 0.039), miR-125b-5p (logFC = −15.02, *p* = 0.043), and miR-122-5p (log FC = −13.81, *p* = 0.049). In the patients in the LC + BM group, two miRNAs were found to be downregulated compared to the HC group: miR-409-3p (logFC = −17.42, *p* = 0.029) and miR-485-3p (logFC = −17.30, *p* = 0.026). The significantly dysregulated miRNAs are shown in Table 2 and Table 3.

### 3.3. Machine Learning Model

To define the classification task, the HC and LC samples were combined and contrasted against the LC + BM group. An initial exploratory analysis, performed on the full dataset, was used to summarize the miRNA features that repeatedly ranked highly in the univariate probe-based selection. This analysis identified eleven miRNAs with relatively high empirical selection frequencies: miR-363-3p, miR-210-3p, miR-194-5p, miR-409-3p, miR-22-3p, miR-2110, miR-326, miR-485-3p, miR-223-5p, miR-16-2-3p, and miR-139-5p. Among these, miR-363-3p, miR-210-3p, and miR-194-5p exhibited the highest stability across the resampling runs. These results are reported for descriptive purposes only; feature selection for predictive modeling was performed independently within each cross-validation training fold, and no fixed set of miRNAs was assumed during the model evaluation.

The predictive performance of all the evaluated models, trained using the nested fea-ture-selection and cross-validation procedure described in Section 2.3, was close to chance level. The areas under the ROC curve were 0.46 for the support vector machine, 0.46 for the random forest, and 0.49 for the elastic net logistic regression model. These results indicate that, under the present modelling framework and data constraints, the selected miRNA features do not provide meaningful discriminative power for the classification task.

**Figure 3:**
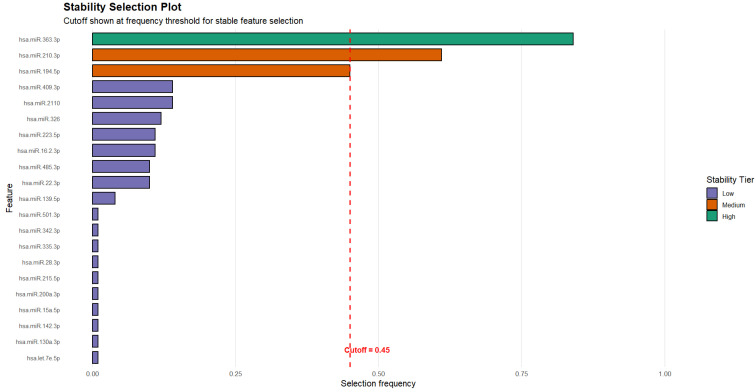
MiRNAs selected for the training of the predictive models with regard to their bootstrap stability. Thresholds for stability tiers were treated as tuning parameters, following Meinshausen et al. [21], who note that results vary little for “sensible” choices of the cutoff within the range 0.6–0.9. Features with stability ≥ 0.70 were therefore classified as highly stable. An exploratory medium-stability tier (≥0.45) was defined below the theoretical range, supported by an elbow in the empirical stability distribution.

**Figure 4:**
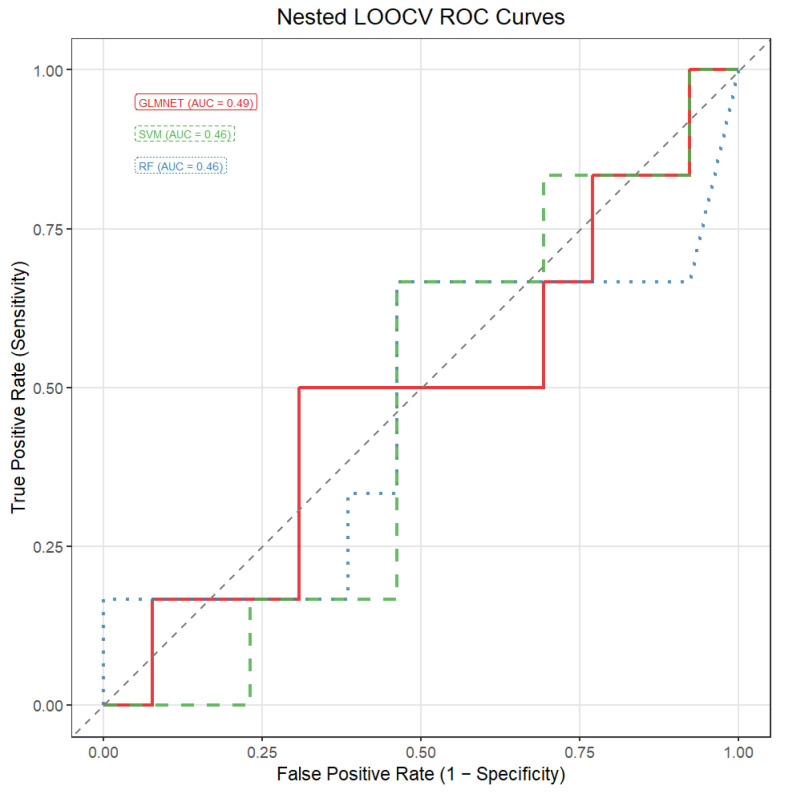
ROC curves for the classification models. GLMNET—logistic regression with elastic net regularization; SVM—support vector machine; RF—random forest; AUC—area under the ROC curve.

Figure 3 illustrates the empirical stability of miRNA features estimated from the full dataset using the probe-based selection procedure. As indicated earlier in this section, this analysis was conducted for exploratory and descriptive purposes only, to visualize the relative robustness of individual miRNAs across repeated resampling runs and was not used for feature selection during model training or performance evaluation, which were performed within a fully nested cross-validation framework. The ROC curves for the respective classification models are displayed in Figure 4.

**Figure 5:**
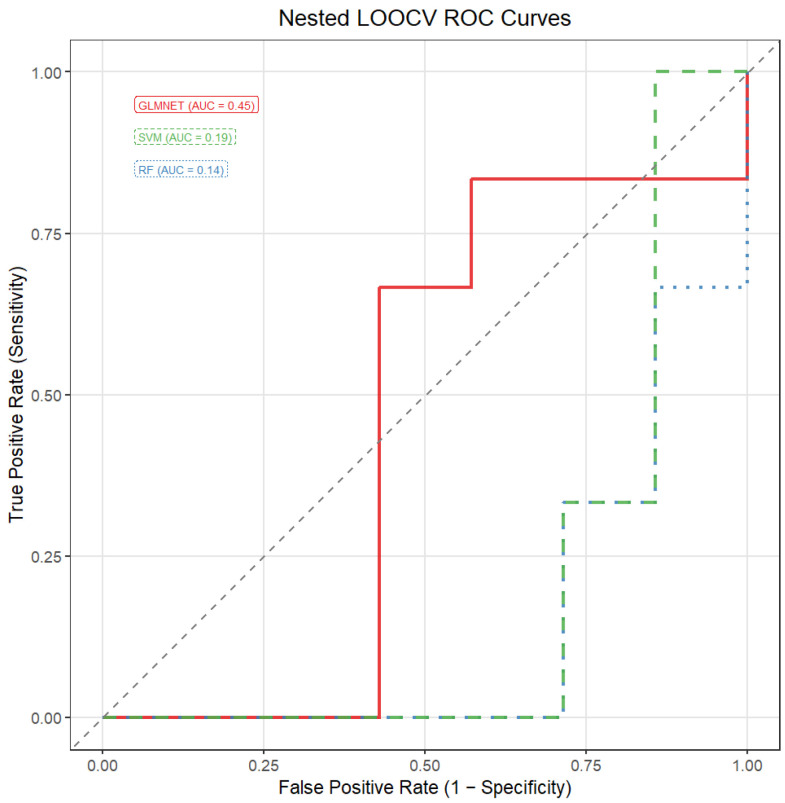
ROC curves for the classification models. Comparison of lung cancer with BM vs. lung cancer without BM groups. GLMNET—logistic regression with elastic net regularization; SVM—support vector machine; RF—random forest; AUC—area under the ROC curve.

**Figure A1:**
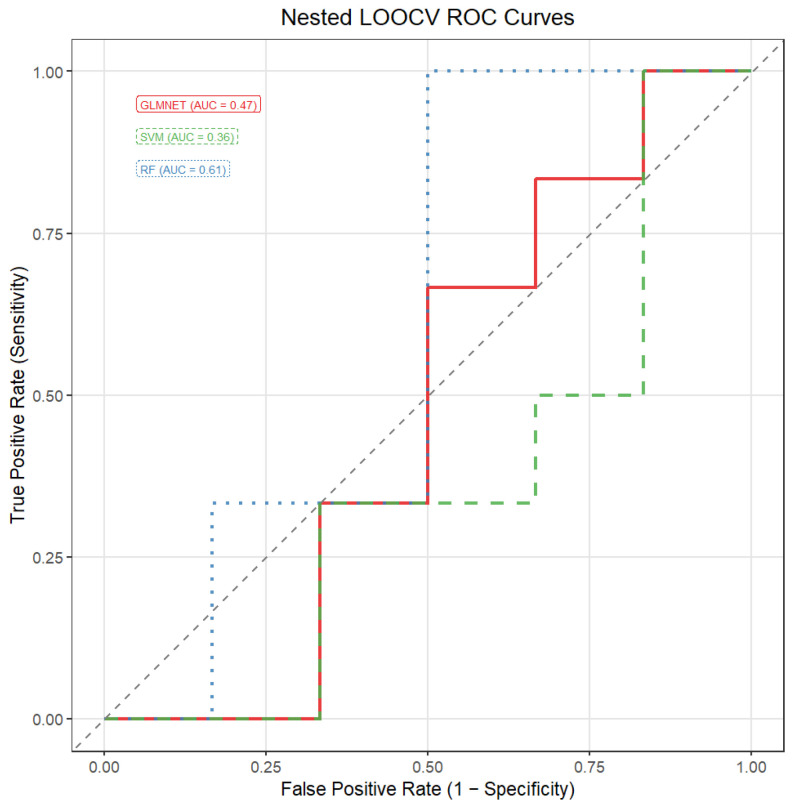
ROC curves for the classification models. Comparison of lung cancer with BM vs. HC groups. GLMNET—logistic regression with elastic net regularization; SVM—support vector machine; RF—random forest; AUC—area under the ROC curve.

Additional subclass-specific analyses were performed in an exploratory manner. No meaningful discrimination under nested cross-validation was observed in the comparison between the patients with lung cancer and brain metastases versus the patients with lung cancer without brain metastases (Figure 5). In the comparison between the LC + BM and the HC groups, modest and model-dependent discrimination was observed (Figure A1 in Appendix B). These findings suggest that the evaluated miRNA panels may capture biological differences but do not yield reliable predictive performance in the present dataset.

## 4. Discussion

miRNAs play an important role in the regulation of gene expression and can contribute to all stages of cancer progression, including tumor growth and local and distant invasion. In this study, we focused on the relationships of miRNAs with brain metastases from lung cancer and their relevance as their potential biomarkers.

We found two miRNAs significantly downregulated in the patients with lung cancer and brain metastases compared to the healthy controls: miR-409-3p and miR-485-3p. These miRNAs exhibited very large negative logFC values, reflecting a pronounced relative downregulation at very low level, near the limit of detection. Although extremely low expression of miRNAs has been reported in the existing literature [22,23], the magnitude of downregulation observed in this study appears even greater. This may result from the small size of the study group, as a limited number of samples with near-threshold expression can disproportionately influence group-level estimates. While this points to the direction of differential expression, the absolute values of the observed fold changes should be interpreted with caution and larger, independent cohorts with sufficient power are required to confirm the extent of the observed downregulation.

To address the primary research objective, a binary classification framework was adopted in which the healthy controls and the patients with lung cancer without brain metastases were combined and contrasted against the patients with lung cancer and brain metastases. This design was motivated by the aim of identifying miRNAs potentially associated with central nervous system metastatic involvement rather than with lung cancer presence alone, and by the need to limit the model complexity given the sample size.

An exploratory feature-ranking analysis performed on the full dataset summarized miRNAs that were repeatedly selected by the probe-based procedure. This analysis identified eleven miRNAs with relatively high empirical selection frequencies: miR-363-3p, miR-210-3p, miR-194-5p, miR-409-3p, miR-22-3p, miR-2110, miR-326, miR-485-3p, miR-223-5p, miR-16-2-3p, and miR-139-5p. Among these, miR-363-3p, miR-210-3p, and miR-194-5p exhibited the highest stability across the resampling runs.

Importantly, these exploratory findings were not used to define a fixed feature set for predictive modeling. Instead, feature selection and model training were repeated independently within each cross-validation training fold. When evaluated under this fully nested framework, the resulting classification models demonstrated performance close to chance level, indicating that the identified miRNAs, while biologically plausible candidates, do not provide sufficient discriminative information to support the reliable prediction of brain metastases in the present dataset.

While this approach is justified by an exploratory setting, it does not fully reflect the most clinically relevant scenario, which is the discrimination of lung cancer patients with brain metastases from those without. While it reduces the risk of overfitting, combining HC and LC groups into a single negative class introduces a heterogeneity in the reference group and may bias performance estimates, therefore limiting the transferability to a clinical setting.

We additionally examined the comparison between the patients with lung cancer and brain metastases and those with lung cancer without metastatic involvement of the brain in an exploratory manner. This subgroup analysis revealed similar patterns of association, with the majority of the analyzed miRNAs present in the initial exploratory feature-ranking analysis. However, when evaluated under a fully nested cross-validation framework, these miRNA panels did not yield reliable discriminative performance, indicating that the observed associations should be interpreted only as hypothesis-generating rather than predictive. This analysis addresses a plausible use-case in which a circulating miRNA profile could support risk stratification for intracranial involvement and help prioritize neuroimaging in patients at higher risk. Such biomarkers would be expected to serve as a complementary tool for neuroimaging techniques, and any clinically useful implementation would require predefined thresholds and evaluation in representative cohorts.

The limited discriminative performance observed across all the evaluated classifiers, i.e., GLMNET, SVM and RF, likely reflects a combination of biological and methodological factors. First, the effective sample size in each cross-validation training fold was small, leading to substantial variance in performance estimates and unstable decision boundaries. Second, although several miRNAs were repeatedly selected in exploratory stability analyses as good discriminators, with miR-363-3p, miR-210-3p, and miR-194-5p being the most highly ranked, stable univariate ranking does not necessarily translate into multivariate separability, particularly when class distributions substantially overlap. Third, the distinction between lung cancer patients with and without brain metastases represents a biologically subtle phenotype that may not be strongly reflected in circulating miRNA profiles alone. Finally, the use of a fully nested cross-validation framework with probe-based feature selection minimizes optimistic bias.

Although the evaluated classifiers included both linear and nonlinear methods, it remains possible that more complex nonlinear models could capture higher-order interactions among miRNAs. However, given the limited sample size and the consistently low performance observed under the nested cross-validation, the present data are not likely to support the reliable training or validation of such models.

miR-409-3p has been extensively studied in recent years, as it was shown to be dysregulated in various malignancies. In most reports, a significant downregulation of miR-409-3p was described [24], suggesting an oncosuppressive role, although its upregulation has also been reported, for instance, in glioblastoma [25]. However, despite some inconsistent results, it was demonstrated to negatively affect the expression of proteins involved in the proliferation, metastasis, and apoptosis of tumor cells. Proposed molecular targets for miR-409-3p, include zinc-finger E-box-binding homeobox-1 (ZEB1) [26] and E74-like factor 2 (ELF2) [27] (identified in osteosarcoma), activating transcription factor 1 (ATF1) in cervical cancer [28], hexokinase 2 (HK2) in colon carcinoma [29], and high-mobility group nucleosome-binding domain 5 (HMGN5) in gliomas [30]. miR-409-3p has also been shown to inhibit the expression of some established prooncogenes, such as c-Met [31,32]. Regarding metastasis induction, a potential connection between miR-409-3p and angiogenesis promoting factors may draw particular attention. In the study by Weng et al. [33], miR-409-3p was found to suppress vascularization and metastasis by downregulating the expression of angiogenin in fibrosarcoma cells. Indeed, studies have also demonstrated the low value of miR-409-3p as a biomarker of metastasis and poor prognosis, including in breast [34], prostate [35], and gastric cancers [36].

The data on the significance of the abnormal expression of miR-409-3p in the central nervous system are scarce. Hu et al. observed that miR-409-3p secreted from activated mast cells via exosomes promoted microglial migration and activation, thereby inducing neuroinflammation by targeting the Nr4a2/NF-κB pathway [37].

miR-485-3p has also been previously proposed as a tumor suppressor agent in several malignancies. In prostate cancer cells, treatment with fludarabine reduced the release of miR-485-3p, suggesting that it was retained by the surviving cells, therefore promoting the expression of pro-survival genes [38]. Its expression levels were also found to be lowered in breast cancer tissues, possibly leading to the overexpression of ZEB1 [39] and PGC-1α, which promote spontaneous metastasis in vivo [40], as well as in osteosarcoma cells, where it is responsible for suppressing oncogenic carboxyl-terminal binding protein 1 (CtBP1) [41] and c-MET and AKT3/mTOR signaling [42]. In glioblastoma multiforme (GBM), miR-485-3p inhibited cell proliferation and migration by silencing ring finger protein 135 (RNF135) expression [43] and was established as an independent predictive factor of worse progression-free survival (PFS) and overall survival (OS) [44]. Finally, the downregulation of miR-485-3p was shown to exert an oncogenic role in NSCLC by acting through a SPI1/SNHG6/miR-485-3p/VPS45 axis [45]. Furthermore, miR-485-3p has been previously linked to neuroinflammation modulation, i.e., in Parkinson’s disease [46] and in Alzheimer’s disease [47].

miR-210-3p has been shown to be dysregulated in various cancers, although its role remains unclear. It was demonstrated that miR-210-3p expression is upregulated in lung cancer tissue and may serve as a potential diagnostic biomarker [48,49]. Some authors reported the induction of miR-210-3p expression under hypoxia, enhancing tumor proliferation, migration, and invasion by downregulating E2F3 expression [50] and by regulating C-C motif chemokine 2 (CCL2) mediated monocyte infiltration [51]. Interestingly, in a study by Zhao et al., miR-210 (along with miR-214 and miR-15a) was included in a three-miRNA signature model to predict the brain metastases of lung adenocarcinoma, which achieved high accuracy [52]. However, there are also some reports indicating the potential oncosuppresive role of miR-210-3p in other cancers, e.g., in a study by Yang et al. [53] it was found to inhibit tumor growth and metastases in bladder cancer in vitro and in vivo.

miR-363-3p has been predominantly characterized as a tumor-suppressive miRNA, acting through the inhibition of cell invasion and EMT. Consistently, miR-363-3p overexpression has been shown to suppress these processes in several malignancies (including lung cancer, colorectal cancer, hepatocellular carcinoma and osteosarcoma) by targeting oncogenic regulators such as NEDD9 [54], proliferating cell nuclear antigen (PCNA) [55] and pseudopodium-enriched atypical kinase 1 (PEAK1) [56]. Among these, sex-determining region Y box 4 (SOX4) has been most consistently identified as a relevant target of miR-363-3p [57,58,59,60]. In a study on lung adenocarcinoma cells, long non-coding RNA X-inactive specific transcript (XIST) bound to miR-363-3p was found to change mouse double minute clone 2 (MDM2) expression and regulate proliferation and apoptosis [61]. However, in glioma cells, miR-363-3p may be overexpressed [62], and suppression of miR-363-3p was shown to inhibit invasion and promote cell death by attenuating EMT and blocking the Wnt/β-catenin pathway [63].

miR-194-5p may be involved in metastatic progression through several distinct mechanisms of action. Similar to miR-363-3p, it is considered as a tumor-suppressive factor, and alterations in its expression may promote EMT by regulating specific targets, including Forkhead Box A1 (FOXA1) [64], ZEB1 [65], TEFM [66] and mitochondrial fission regulator 1 (MTFR1) [67]. Interestingly, in mouse models, miR-194-5p upregulation restrained angiogenesis and increased tight junction protein levels in angiotensin II-treated human retinal microvascular endothelial cells via the inactivation of SOX17/VEGF signaling [68]. In another report, the regulation of circ-USP1 via the miR-194-5p/FLI1 axis mediated the regulation of tight junction proteins, causing the disruption of blood–tumor barrier permeability in gliomas [69]. In addition, some hypotheses suggest that miR-194-5p may influence the regulation of proinflammatory cytokine secretion in the central nervous system via the neurexophilin 1 pathway [70], which could play a role in the stabilization of a metastatic niche within the brain. It may be further strengthened by the observation that the sponging of miR-194-5p promoted the PD-L1-mediated immune escape of lung cancer cells [71]. miR-194-5p was also linked to the development of brain metastases in breast cancer by regulating the expression of myocyte enhancer factor 2C (MEF2C), which was hypothesized to facilitate the crosstalk between tumor cells and astrocytes [72]. In the study by Roskova et al. [73], miR-194-5p was included in a panel that effectively distinguished the primary tumor origin in cases of brain metastases.

miR-22-3p has also been associated with NSCLC as a potential tumor suppressor. In one study [74], the expression of miR-22-3p was downregulated in lung adenocarcinoma and negatively correlated with established pro-metastatic factors, such as vascular endothelial growth factor (VEGF), marked microvessel density (MVD), and matrix metalloproteinases (MMP-3 and MMP-7). In another publication [75], the overexpression of miR-22-3p suppressed the migration and the epithelial–mesenchymal transition (EMT) of NSCLC cells by targeting RAS-related C3 botulinum toxin substrate 1 (RAC1). In an experimental setting, miR-22-3p mimics the inhibited MET-STAT3 signaling and induced apoptosis in lung adenocarcinoma cells, which may be significant with regard to metastatic niche colonization [76].

Data on the role of miR-2110 in cancer progression is limited. Available reports showed the possible effect of miR-2110 on the expression of minichromosome maintenance protein 2 (MCM2), which is involved in cancer cell proliferation [77].

Similar to the aforementioned miRNAs, miR-326 exhibits pleiotropic effects, influencing several potential mechanisms associated with metastasis. The potential molecular targets of miR-326 include ZEB1 [78], a transcription factor involved in EMT and the tropism of immunosuppressive cells and chemokines, possibly leading to the formation of a tumor immunosuppressive microenvironment [79]. In lung cancer, miR-326 has been shown to regulate several key pathways involved in tumor invasion, including Krüppel-like factor 3/specificity protein 1 (Sp1/KLF3) [80], beclin 1 (BECN1) [81], cyclin D1 and Bcl-2 [82], CXC Motif Chemokine Receptor 5 (CXCR5) [83] and nucleosome-binding protein 1 (NSBP1) [84]. Moreover, miR-326 has been reported to affect the regulation of SLC7A11, a protein promoting tumor aggressiveness and suppressing ferroptosis in NSCLC cells [85]. Another molecular target of miR-326, ADAM17 was reported to influence the expression of EMT markers [86]. Similar to miR-194-5p, miR-326 also modulates pathways associated with PD-L1, which may impact not only the survival of tumor cells within the metastatic niche but also the development of resistance to targeted therapies [87,88]. An analysis by Sun et al. [89] proved that in NSCLC, miR-326 inhibited the matrix metalloproteinases MMP-7 and MMP-9.

Interestingly, there is emerging evidence suggesting the role of miR-326 in the pathogenesis of neuroinflammatory diseases, particularly multiple sclerosis, where it has been proposed to contribute to TH17 differentiation and maturation [90,91].

In one report, the overexpression of miR-223-5p remarkably suppressed the proliferation of NSCLC cells in vitro and in vivo and led to reduced migration and invasion in NSCLC cells [92]. Similar to miR-326, the inhibition of miR-223-5p promoted SLC7A11 expression and resulted in ferroptosis suppression in NSCLC cells [93].

Despite the limited data available in lung cancer, miR-16-2-3p is considered an oncosuppressive miRNA. In other cancers, its influence on primary tumor growth, metastasis seeding, chemoresistance, and invasiveness has been reported through the modulation of the expression of targets such as fibroblast growth factor receptor 2 (FGFR2) [94]. In bladder cancer, miR-16-2-3p was found to influence the expression of the Wnt5a/ZEB1 axis, therefore preventing EMT [95].

miR-139-5p acts as a tumor suppressor and is known to be downregulated in lung adenocarcinoma [96]. In NSCLC, the decreased expression of miR-139 was significantly associated with distant lymph node metastasis and histological invasiveness [97], as well as lytic bone metastasis [98]. miR-139-5p has been primarily associated with the regulation of factors involved in EMT. One of its most strongly linked molecular targets is ZEB1, described in lung cancer and in other malignancies [99,100,101]. Additional targets which were proposed in the literature include matrix metalloproteinase 2 (MMP2) [102], ATPase Family AAA Domain Containing 2 (ATAD2) [103], and cyclin D2 [104]. Moreover, the upregulation of miR-139-5p can inhibit NSCLC proliferation, migration, and invasion in vitro via targeting insulin-like growth factor 1 receptor (IGF1R) [105,106]. It was shown that endothelial CXC Motif Chemokine Receptor 4 (CXCR4) is negatively regulated by miR-139-5p [107], which may be important in the context of tumor-related angiogenesis. The association between miR-139-5p and CXCR4 was later confirmed in squamous cell carcinoma [108].

**Figure 6:**
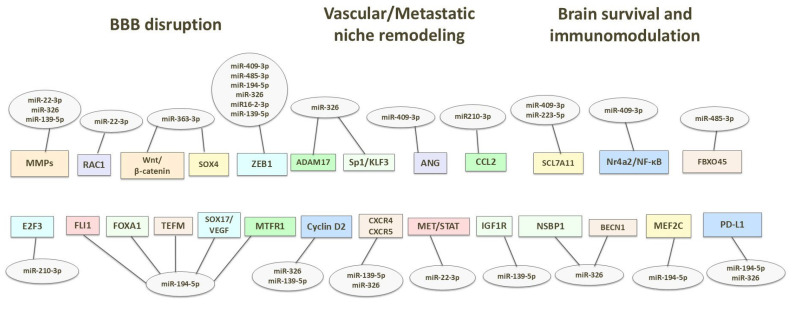
Potential molecular targets of the analyzed miRNAs.

Despite the limited evidence directly linking miRNA profiles to the risk for brain metastases from lung cancer, our study provides new insights into the potential role of miRNA expression in this field. The miRNAs identified as relevant in our study may exert both oncogenic and oncosuppressive effects, and some of them are also involved in neoplastic or inflammatory processes within the nervous tissue. It is worth emphasizing that the CNS represents a distinct metastatic site, separated by the blood–brain barrier (BBB), which dynamically regulates the transfer of particles and cells from the systemic circulation. Therefore, the mechanisms of the metastasis to the brain may be unique and more complex in comparison to other sites of cancer spread. These mechanisms may include promoting the transmigration of circulating tumor cells across the blood–brain barrier by impairing the integrity of brain microvascular endothelial cells (BMECs) and altering vascular structure and function (i.e., angiogenesis and increased small vessels permeability), processes supporting the establishment of a premetastatic microenvironment and the adaptation/survival of tumor cells within the brain tissue [109,110]. Figure 6 shows potential molecular targets of the analyzed miRNAs, consistent with their involvement in the spread of cancer to the CNS.

Although the available data in the literature are limited, some other miRNAs have also been linked to CNS involvement in lung cancer and were suggested to regulate metabolic pathways directly associated with the unique characteristics of brain metastases. Li et al. [111] reported the modulating effect of miR-596-3p on YAP1 and IL-8, factors known to inhibit the invasion of cancer cells and the permeability of the BBB. YAP1 was also shown to be regulated by miR-550a-3-5p, which was significantly enriched in brain metastatic exosomes in lung cancer [112]. Among the miRNAs potentially promoting metastasis by modifying the BBB, there are also miRNA-522-3p [113] and miR-1207-5p [114]. miR-21, similar to the miR-22-3p revealed in our study, may influence apoptosis mechanisms in the metastatic niche via STAT3 [115]. Moreover, several other miRNAs were found to be dysregulated in lung cancer with CNS involvement, such as miRNA-197 and miRNA-184 [116], miRNA-328 and miRNA-378 [117], miR-328 [118], mmiR-330-3p [119] and miR-375 [120]. Tsakonas et al. [121] developed a panel of 11 miRNAs that differentiated primary NSCLC and brain metastases based on tissue biopsies. However, the particular mode of action of these miRNAs has not been defined. The heterogeneity of the cited findings likely reflects the diversity in the type of biological material analyzed (tissue vs. serum/plasma), the patient selection, the disease stage, and the methodological approaches. A lack of clear conclusions from these studies emphasizes the need for further investigation.

In this study, we assessed serum miRNA expression profiles in lung cancer patients with and without metastases to the CNS and compared them to healthy subjects. In order to maximize the chance of identifying potential biomarkers differentiating patients with brain metastases in relatively small groups of participants, we decided to use an exploratory approach using a broad panel of 179 specific miRNAs instead of focusing on a previously selected narrow set of molecules. We believe that the use of a broad miRNA panel allows for a hypothesis-generating purpose of the analysis and for the detection of candidate miRNAs that can be validated in larger, independent cohorts. Such a methodology minimizes the risk of omitting miRNAs potentially important for the metastatic progression of the disease, especially given the fact that there is still a knowledge gap regarding the role of miRNAs in CNS neoplastic involvement. Another strength of this study is associated with the application of up-to-date analytical techniques, including machine learning algorithms, which allowed a more accurate identification of complex associations between the selected variables.

We believe that these preliminary results encourage further investigation on miRNA profiles as putative biomarkers of CNS involvement in lung cancer. The expected clinical benefits from applying such a predictive tool would include the early identification of patients at increased risk for brain metastases and an individualized approach to their management (intensive treatment strategies and regular follow-up).

Nevertheless, several limitations need to be acknowledged. The sample size was relatively small, which might have substantially affected the robustness and generalizability of the results. The restricted size of the study group precluded the use of an independent external validation set. Therefore, all the obtained results are interpreted purely exploratorily as indicators of a potential signal rather than validated diagnostic tools.

Additional potential limitations include preanalytical factors that may have influenced the miRNA stability and measured expression levels, such as differences in the duration of sample storage prior to the analysis. Serum storage at −20 °C (rather than −80 °C) may also represent a preanalytical limitation that could contribute to measurement variability. Although all the samples were processed according to standardized protocols, such sources of technical variability cannot be fully excluded and may have contributed to the observed differences, especially given the small groups.

In addition, given the limited number of cases, it was not possible to perform a reliable analysis according to the different histological subtypes of lung cancer, which may exhibit distinct molecular and metastatic profiles. Furthermore, the LC + BM group tended to have a more advanced disease at baseline, which may have partially contributed to the observed miRNA expression differences. The LC versus LC + BM contrast was not fully clinically homogeneous. The LC group included patients with metastases to the other lung, while the LC + BM group exhibited additional extracranial metastatic disease (liver and adrenal gland). Due to the limited sample size, sensitivity analyses restricted to clinically more homogenous subgroups were not feasible. A possible oncologic treatment-related variability in miRNA expression may also exist. The serum sampling at admission occurred at variable time points relative to the diagnosis and the treatment initiation, which could have influenced the circulating miRNA levels. Therefore, the observed miRNA differences may partially reflect the global metastatic burden and treatment-related effects. Larger, clinically stratified cohorts with standardized sampling and adjustment for potential confounding factors are required for confirmatory evaluation. Nevertheless, the type of analysis applied in this study was supposed to reveal robust miRNA signals that persist regardless of therapeutic context.

## 5. Conclusions

In this study, we found differences in the expression of particular miRNAs and reviewed candidate miRNAs potentially associated with the development of brain metastases in lung cancer. However, under a fully nested framework, the analyzed classification models in the present dataset did not demonstrate sufficient discriminative information to support a reliable prediction of brain metastases.

While our analysis provides only exploratory insights, the findings should be interpreted with caution and require validation in larger, independent cohorts before any clinical or translational implications can be established. Therefore, there is a need for further studies focusing on establishing causal associations within the molecular pathways underlying central nervous system involvement in lung cancer.

## Figures and Tables

**Table 1:** Characteristics of the study group. BM—brain metastasis; F—females; M—males.

	Lung Cancer Without BM *n* = 7	Lung Cancer with BM *n* = 6	Healthy Controls *n* = 6
Gender	4 F, 3 M	4 F, 2 M	4 F, 2 M
Age	66.1 ± 7.4	63.2 ± 8.8	67.8 ± 10.7
Smoking (active)	1 (14.3%)	2 (33.3%)	0
TNM staging:			
T1	2 (28.6%)	1 (16.7%)	-
T2	3 (42.9%)	2 (33.3%)	-
T3	1 (14.3%)	-	-
T4	1 (14.3%)	3 (50%)	-
N0	6 (85.7%)	-	-
N1	-	1 (16.7%)	-
N2	1 (14.3%)	3 (50%)	-
N3	-	2 (33.3%)	-
M0	5 (71.4%)	-	-
M1	2 (28.6%)	6 (100%)	-
Distant metastases:			
Brain	-	6 (100%)	-
Other lung	2 (28.6%)	1 (16.7%)	-
Adrenal gland	-	2 (33.3%)	-
Liver	-	1 (16.7%)	-

**Table 2:** Significantly dysregulated miRNAs in patients with lung cancer without brain metastases compared to healthy controls. FC—fold change.

miRNA	FC	logFC	*p*-Value
miR-328-3p	0.000011	−16.51	0.005
miR-92a-3p	0.001422	−9.46	0.008
miR-101-3p	0.000041	−14.57	0.021
miR-451a	0.003440	−8.18	0.027
miR-374a-5p	0.000018	−15.74	0.028
miR-100-5p	0.000005	−17.61	0.036
miR-197-3p	0.000019	−15.71	0.039
miR-125b-5p	0.000030	−15.02	0.043
miR-122-5p	0.000070	−13.81	0.049

**Table 3:** Significantly dysregulated miRNAs in lung cancer patients with brain metastases compared to healthy controls. FC—fold change; logFC—second-degree logarithm of FC.

miRNA	FC	logFC	*p*-Value
miR-409-3p	0.000006	−17.42	0.029
miR-485-3p	0.000006	−17.30	0.026

## Data Availability

The datasets analyzed during this study are available from the corresponding author on reasonable request.

## Abbreviations

The following abbreviations are used in this manuscript:

LC	Lung cancer
miRNAs	MicroRNAs
NSCLC	Non-small cell lung cancer
SCLC	Small cell lung cancer
HC	Healthy control
BM	Brain metastasis
CNS	Central nervous system
OS	Overall survival
LR	Logistic regression
SVM	Support vector machine
RF	Random forest

## Appendix A

**Table A1:** Detailed study group characteristics.

Study Group	Gender (F—Female, M—Male)	Age (Years)	Histological Type of LC	TNM	Time From Diagnosis (Months)	Site of Metastases	Comorbidities	Current Smoking Status	Treatment	Treatment status
Lung cancer without BM										
LC1	F	71	NSCLC	T1N0M0	1		AH, HFrEF	No	Surgical	Surgical treatment 1 month prior to sampling
LC2	F	54	NSCLC	T4N2M1	18	other lung	AH, anemia	No	Immunotherapy, CTH	Active (systemic therapy 1 month prior to sampling)
LC3	F	73	NSCLC	T2N0M0	1		AH, asthma/COPD, hypothyroidism	No	Surgical	Surgical treatment 1 month prior to sampling
LC4	F	64	NSCLC	T3N0M1	36	other lung	AH, DM2, obesity	No	Immunotherapy	Active (systemic therapy 1 month prior to sampling)
LC5	M	61	NSCLC	T2N0M0	1		BPH	No	Surgical	Surgical treatment 1 month prior to sampling
LC6	M	65	NSCLC	T2N0M0	1			Yes	Surgical	Surgical treatment 1 month prior to sampling
LC7	M	75	NSCLC	T1N0M0	3		COPD, AH, obesity	No	Surgical, CTH	Active (systemic therapy 1 month prior to sampling)
Lung cancer with BM										
LCBM1	F	71	NSCLC	T4N2M1	2	brain, liver	AH	No	CTH, RTH	Treatment initiated after sampling
LCBM2	F	53	NSCLC	T4N2M1	1	brain, adrenal gland	AH, anemia	No	CTH, RTH	Treatment initiated after sampling
LCBM3	F	58	NSCLC	T1N2M1	1	brain	Alcohol use disorder	No	Surgical (palliative)	Treatment initiated after sampling
LCBM4	F	75	NSCLC	T2N3M1	1	brain	AH	Yes	Palliative care	Treatment initiated after sampling
LCBM5	M	56	NSCLC	T2N3M1	1	brain, adrenal gland		Yes	Palliative care	Treatment initiated after sampling
LCBM6	M	66	NSCLC	T4N1M1	13	brain, other lung	benign hyperthyroidism	No	CTH, RTH	Systemic therapy in the past (more than 6 months prior to sampling)
Healthy controls										
HC1	F	79						No		
HC2	F	71					AF, obesity	No		
HC3	F	54						No		
HC4	F	73					AH	No		
HC5	M	55						No		
HC6	M	75					AH	No		
